# Recombinase-based amplification coupled with lateral flow chromatography for the specific and sensitive detection and identification of *Leishmania major* in cutaneous leishmaniasis patients

**DOI:** 10.3389/fmicb.2024.1514684

**Published:** 2025-01-27

**Authors:** Insaf Bel Hadj Ali, Yusr Saadi-Ben Aoun, Imen Khammeri, Hejer Souguir, Emna Harigua-Souiai, Hamed Chouaieb, Ahmed S. Chakroun, Meryem Lemrani, Aicha Kallel, Kalthoum Kallel, Nabil Haddad, Oussaima El Dbouni, Rhea N. Coler, Steven G. Reed, Akila Fathallah-Mili, Ikram Guizani

**Affiliations:** ^1^Laboratory of Molecular Epidemiology and Experimental Pathology-LR16IPT04, Institut Pasteur de Tunis, University of Tunis El Manar, Tunis, Tunisia; ^2^Parasitology Department, UH Farhat Hached, Faculty of Medicine of Sousse, University of Sousse, Sousse, Tunisia; ^3^Institut Pasteur du Maroc, Casablanca, Morocco; ^4^Parasitology Department, UH La Rabta, Faculty of Medicine of Tunis, Tunis, Tunisia; ^5^Faculty of Public Health, Lebanese University, Beirut, Lebanon; ^6^Rafic Hariri Hospital, Beirut, Lebanon; ^7^Center for Global Infectious Disease Research, Seattle Children’s Research Institute, Seattle, WA, United States; ^8^Department of Pediatrics, University of Washington School of Medicine, Seattle, WA, United States; ^9^Department of Global Health, University of Washington, Seattle, WA, United States; ^10^HDT Bio, Seattle, WA, United States

**Keywords:** cutaneous leishmaniases, molecular diagnosis, RPA/RAA, *Leishmania major*, lateral flow chromatography, point of care diagnosis

## Abstract

**Introduction:**

Cutaneous leishmaniases (CL), a wide range of cutaneous diseases caused by diverse species of *Leishmania* genus parasites, are among the most neglected infectious diseases. While they are non-fatal, CL are highly morbid with disfiguring lesions, which could be chronic, leaving lifelong unsightly scars; they are combined with psychological distress and social stigma. The efficiency of treatment highly depends on the infecting *Leishmania* species. Diagnosis is mainly based on microscopic direct examination (DE) of Giemsa-stained smears needing experienced microscopists. It can be laborious and time-consuming when the parasite load is low. DE is poorly sensitive and does not identify *Leishmania* species. So far, only DNA assays accurately identify the species. Despite their wide use for generic detection, PCR methods also require equipment and additional steps to identify causal *Leishmania* species. *L. major* is hyperendemic in many countries in Africa, the Middle East, and Asia, where other species co-occur with different endemicity levels according to the situations. This complicates disease management and treatment, particularly as distribution and epidemiology of leishmaniases remain poorly understood. Here, we aimed for a simple and rapid molecular diagnostic test to detect and identify *L. major*, a predominant CL causal species, which could be prone to become a control tool at the point of care, in endemic areas, using isothermal recombinase DNA amplification (recombinase polymerase amplification, RPA, or recombinase aided amplification, RAA) coupled to detection by the lateral flow (LF) chromatography on a PCRD cassette.

**Methods:**

To develop an *L. major* species-specific RPA-LF assay, computational analysis of 70 *Leishmania* DNA targets, identified through bibliography and database searches, selected five targets. We designed and tested 7 primer pairs/probe sets to specifically amplify *L. major* DNAs. First, the primers were tested for species specificity and sensitivity using basic RPA chemistry. Then, to develop RPA-coupled LF detection, we shifted to the nfo chemistry.

**Results:**

This way, we retained one set for further investigation, which confirmed it is *L. major* species-specific. Tested on 86 human cutaneous samples, this selected set was able to detect 100% of *L. major* infections in confirmed CL patients. We did not observe any cross-reactivity with lesions due to *L. infantum* or *L. tropica*.

## Introduction

Cutaneous leishmaniases (CL) constitute a wide range of cutaneous vector-borne diseases caused by diverse species of *Leishmania* genus parasites. This group represents one of the most neglected infectious diseases. In Old World, more than 1 million CL cases are reported each year, of which 80% occur in the Middle East and North Africa (MENA) region. Moreover, the MENA is at increased risk of emergence and epidemics due to the changes in epidemiological trends related to environmental or climate changes, migrations, or conflicts (reviewed by Tabbabi, 2019). Indeed, CL in MENA, caused by four species (*L. major*, *L. tropica*, *L. infantum*, and *L. donovani*), have complex epidemiology due to species diversity and co-endemicity and even co-sympatry, their different levels of prevalence, microfocal distribution, and many unknowns in transmission cycles.

Zoonotic cutaneous leishmaniasis (ZCL) due to *L. major* is endemic in all countries in the MENA region (reviewed by Tabbabi, 2019). It has diverse clinical manifestations and is known to be epidemic to hyperendemic in these countries while the transmission of other species encountered is less frequent to sporadic, with exceptions of Syria or Sudan where *L. tropica* and *L. donovani* are highly endemic respectively, and Morocco where *L. tropica* is increasingly prevalent (El Idrissi Saik et al., 2022). Furthermore, while several Sub-Saharan African countries face gaps in identifying the etiology of *Leishmania* species responsible for CL, others, such as Burkina Faso, Nigeria, Niger, and Cameroon, have reported *L. major* as the sole species causing CL in these regions (reviewed by Blaizot et al., 2024), a statement that may be revised by the increasing use of DNA assays for species detection and identification.

While CL is not fatal, it is highly morbid with disfiguring and/or lifelong scars leading to social stigma and psychological distress (Chahed et al., 2016; Elfaki et al., 2024). Furthermore, CL was recently called “the great imitator” (Gurel et al., 2020) as it could mimic a wide range of skin diseases, which makes it difficult to diagnose on the sole base of clinical manifestation. CL diagnosis is mainly done by microscopic direct examination (DE) of smears of Giemsa-stained lesion that requires experienced microscopists, time-consuming, and laborious when parasite loads are low. DE is 100% specific but is poorly sensitive (43%) (Mouttaki et al., 2014) and does not identify *Leishmania* species given the similar morphology of their amastigote forms. Only molecular DNA assays accurately identify the species. Etiologic diagnosis of CL is crucial as it is known that treatment protocols depend on the species (Arevalo et al., 2007; Mosimann et al., 2013); efficiency of CL treatment is also highly dependent on *Leishmania* species (Madusanka et al., 2022).

Several PCR-based methods have been developed for the specific and sensitive detection of *Leishmania* parasites responsible for CL (Table 1). These include conventional PCR assays (Rostami et al., 2020), real-time PCR, PCR-RFLP, and PCR sequencing, among others. While these methods can detect *Leishmania* parasites, species identification often requires complementary techniques, such as RFLP (Schönian et al., 2003; Bel Hadj Ali et al., 2021) or sequencing (Stevenson et al., 2010), to enable species identification. Real-time PCR is among the most highly sensitive methods for detecting *Leishmania* DNA (Stevenson et al., 2010; Nath-Chowdhury et al., 2016). However, the requirement for specialized equipment, such as real-time PCR machines, can limit its accessibility in resource-limited settings.

**Table 1:** Commonly used methods for CL diagnosis: examples of performances.

	Se (%)	Sp (%)	References
DE	43	100	Mouttaki et al. (2014)
Culture	29	100	Mouttaki et al. (2014)
kDNA PCR	100	71	Mouttaki et al. (2014)
ITS1 PCR	90–100	93–100	Mouttaki et al. (2014) and Al-Rashed et al. (2022)
18S qPCR	90.9	81.8	Filgueira et al. (2020)
hsp70 qPCR	83.6	60.6	Filgueira et al. (2020)
*L. infantum* DNA repeat region real-time RPA	100	100	Mondal et al. (2016)
kDNA LAMP	48.4–86.7	87.9–100	Soares et al. (2024) and Taye et al. (2024)
m22-RPA/LF	100	88	This study

DE, direct examination; Se, sensitivity; Sp, specificity.

In low-resource or remote settings where there is a lack of equipped laboratories, molecular diagnostics with a point-of-care (POC) format would guarantee an accurate diagnosis and equitable and better access of patients to health care wherever they are. Actually, healthcare challenges in such settings mainly relate to infrastructure and budget (Maphumulo and Bhengu, 2019). This emphasizes the importance of point-of-care molecular diagnostics for prevention, timely patient management, and disease control.

Over the last decade, the POC format has been given a special attention, and a broad spectrum of technologies, reagents, test kits, and equipment were developed (Kozel and Burnham-Marusich, 2017). With the advent of PCR as the gold standard technology in molecular diagnostics for pathogen detection, a range of DNA amplification technologies have been developed to expand their use at the POC, thus allowing to address the need for POC CL testing, requiring minimal equipment while being fast in delivering DNA products. The most commonly used are the loop-mediated isothermal amplification (LAMP) (Erber et al., 2022; Soares et al., 2024; Taye et al., 2024) and the recombinase-based amplification [recombinase polymerase amplification (RPA) or recombinase-aided amplification (RAA)] (Bharadwaj et al., 2021; Khan et al., 2021; Travi et al., 2021; Mondal et al., 2016). RPA/RAA is an isothermal DNA amplification technology with a simple experimental design, by contrast to LAMP that requires four to six primer pairs, to amplify one DNA target. Indeed, RPA operates at low temperatures (37°C–42°C) and requires only two primers or two primers and a probe depending on the used chemistry (Piepenburg et al., 2006). RAA is another designation for this technology (Xue et al., 2020). Using RPA or RAA (according to the kit supplier) to develop molecular diagnostics potentially overcomes current limitations of cost, complexity, and resources while ensuring rapidity, efficiency, and accuracy of the results (Ghosh et al., 2021).

In this study, we aimed to develop a simple, rapid sensitive, and cost-effective *L. major*-specific DNA-based assay. The test based on an isothermal recombinase-based amplification coupled to lateral flow immuno-assay detection would contribute to diagnosing *L. major*-infected patients or their screening in remote areas.

## Materials and methods

### Ethical statement

The study was conducted after obtaining the ethical approval from Ethic Committees of: Institut Pasteur de Tunis (Ref:2016/24/I/LRIPT04), Rafic Hariri University Hospital Lebanon (Ref: INV-2017-324) and Faculty of Medicine and Pharmacy University Mohamed V Rabat, Morocco Faculty of Medical Sciences (Ref: 51/17). All study participants provided signed informed consent. The collected samples and data were codified and treated anonymously.

### *Leishmania* parasites

Sequencing surveys and RPA assays were set up and optimized using characterized strains belonging to *L. major* (*N* = 14), *L. infantum* (*N* = 11), *L. donovani* (*N* = 5) *L. tropica* (*N* = 11), and other Old World *Leishmania* species including *L. aethiopica* (*N* = 1), *L. arabica* (*N* = 1), *L. turanica* (*N* = 1), and the *Sauroleishmania L. tarentolae* (*N* = 1). The corresponding strains were isolated from a range of hosts including human cases, sandflies, and reservoirs from various geographical origins in Africa, the Middle East, or Asia. Tunisian and Sudanese strains were obtained from field studies or health centers in Tunisia and Sudan. All the other strains were obtained from reference centers in Montpellier, London, or Rome. Details describing these strains are shown in Table 2.

**Table 2:** Description of the studied strains.

WHO code	Strain	Pathology	Species	Zymodem
MPSA/TN/87/Ron44*	R44	NA	*L. major*	MON-25
MPSA/TN/87/Ron99*	R99	NA	*L. major*	MON-25
MPSA/TN/87/Ron102	R102	NA	*L. major*	MON-25
MPSA/TN/87/Ron 114*	R114	NA	*L. major*	MON-25
MPSA/TN/ 87/Ron155*	R155	NA	*L. major*	MON-25
MPSA/TN/89/Psa1	Psa1	NA	*L. major*	NT
MPSA/TN/89/Psa5	Psa5	NA	*L. major*	NT
ISAL/IN/73/STDBM*	STDBM	NA	*L. major*	LON-6
MHOM/IL/80/Friedlin*	L3171	CL	*L. major*	MON-103
MHOM/IL/83/IL32*	IL32	CL	*L. major*	MON-68
MHOM/IL/67/Jericho II	Jericho II	CL	*L. major*	MON-26
MHOM/IL/83/IL24*	IL24	CL	*L. major*	MON-66
MHOM/IL/83/IL53*	IL53	CL	*L. major*	MON-67
MRHO/SU/59/P-Strain*	Pstrain	NA	*L. major*	MON-4
MHOM/GR/00/LA28*	LA28	CL	*L. tropica*	LON-16
MHOM/IQ//73/Bumm30*	Bumm30	VL	*L. tropica*	LON-17
MHOM/IQ/76/BAG 17	Bag17	CL	*L. tropica*	LON-24
MHOM/IQ/73/A Sinaï III*	Ass III	CLR	*L. tropica*	LON-11
MCAN/IN/71/DBKM*	DBKM	NA	*L. tropica*	MON-62
MRAT/IQ/73/Adhanis I	Adhanis	NA	*L. tropica*	MON-5
MHOM/IQ/76/BAG 9*	BAG 9	CL	*L. tropica*	MON-53
MHOM/IQ/65/L75*	L75	CL	*L. tropica*	MON-6
MHOM/SU/74/SAF K27*	K27	CL	*L. tropica*	MON-60
MHOM/TN/06/AM*	AM	CL	*L. tropica*	NT
MHOM/TN/09/Leep0920*	Leep0920	CL	*L. tropica*	NT
MHOM/IL/00/Gabaï159*	Gabai 159	CL	*L. tropica*	LON-9
MHOM/IL/78/Rachnan*	Rachnan	CL	*L. tropica*	MON-60
MHOM/TN/80/IPT1*	IPT1	VL	*L. infantum*	MON-1
MHOM/TN/94/VL49*	LV49	VL	*L. infantum*	MON-24
MHOM/TN/96/Drep08	Drep 08	CL	*L. infantum*	MON-1
MHOM/TN/98/Drep16	Drep 16	CL	*L. infantum*	MON-24
MCAN/TN/89/Alm220	ALM220	CanL	*L. infantum*	NT
MHOM/TN/88/Aymen	AYMEN	VL	*L. infantum*	MON-1
MHOM/TN/88/Nabil	NABIL	VL	*L. infantum*	MON-1
MHOM/TN/92/LV08*	LV08	VL	*L. infantum*	NT
MHOM/TN/93/LV10*	LV10	VL	*L. infantum*	MON-80
MHOM/TN/88/KA 439	KA439	VL	*L. infantum*	MON-1
MHOM/TN/94/LV50*	LV50	VL	*L. infantum*	MON-1
MHOM/TN/97/Drep13*	Drep 13	CL	*L. infantum*	MON-24
MHOM/TN/97/Drep14*	Drep 14	CL	*L. infantum*	MON-24
MHOM/TN/97/Drep15*	Drep 15	CL	*L. infantum*	MON-24
MHOM/TN/96/Drep5*	Drep 05	CL	*L. infantum*	MON-1
MHOM/SD/00/MW111*	MW111	VL	*L. infantum*	MON-30
MHOM/ET/67/HU3	L698	VL	*L. donovani*	MON-18
MHOM/ET/72/L100	L100	CL	*L. aethiopica*	MON-14
MPSA/SA/84/Jisha238	Jisha238	NA	*L. arabica*	MON-64
MRHO/SU/74/95A	95A	NA	*L. turanica*	MON-64
IMIN/IT/86/MIN1	MinI	NA	*L. tarentolae*	–

*Strains used for sequencing surveys and primers/probe design validation.

CL, cutaneous leishmaniasis; VL, visceral leishmaniasis; NT, not typed; NA, not applicable.

–, unknown.

### Collection of study sites, data, and clinical samples

The sample and data collection were undertaken in different study sites, including Tunisia, Lebanon, and Morocco. In Tunisia, we collected, in total, 55 samples from informed and consented patients referred for routine diagnosis to the parasitology departments of Farhat Hached Hospital in Sousse (*N* = 49) and La Rabta Hospital in Tunis (*N* = 6). Patient recruitment in these departments mainly reflect the different levels of CL endemicity and distribution of parasites in the different regions of Tunisia (Central vs. Northern Tunisia). In Lebanon, we collected eight CL samples from consented patients having consulted at the Rafic Hariri Hospital; two of whom were Lebanese, and the six others were Syrian refugees. In Morocco, we conducted a field study in the Azilal Province and Ouarzazate/Zagora where we collected samples from 23 consented patients. Patients were sampled by dermal scraping of the lesions in Tunisia and Morocco, and by swabs of lesions following lesion scrapping in Lebanon. Demographic and clinical data of the collected samples are provided in Supplementary Table S1. It also includes the country or city of patient recruitment. The nucleic acid extraction method was harmonized by using the QIAamp DNA Kit (Qiagen, Germany) at the different study sites. We used microscopic direct examination as the reference method for parasitological diagnosis by the visual detection of amastigotes. We followed the criteria suggested by the WHO (WHO, 2010) for the parasitic load classification reported in Supplementary Table S1. Detection and identification of etiological agents in lesions were carried out by ITS1 PCR-*HaeIII* RFLP as described by Schönian et al. (2003). Epidemiological and clinical data were collected using a harmonized questionnaire that was digitalized as described by Preprint Harigua et al. (2020).

### Target identification and screening

We used different approaches to identify species-specific targets for the recombinase amplification-based assays. First, we used a set of *Leishmania* coding sequences (CDS) in a range of species, available in our laboratory, as potential targets. Then, a second screen included a bibliography search of known targets described by other studies for their species specificity (Rogers et al., 2011) and species differentiation ability (Zelazny et al., 2005). The third screen included a computational screening of all known CDS in the *L. major* genome against the whole genomes of *L. infantum* and *L. tropica* available in the public databases selecting those that presented less than an 80% similarity rate. Within all the studied targets, those presenting high interspecies polymorphism rates or sequence gaps were retained for the design of RPA primers.

### RPA primer design and validation by a sequencing survey

We designed RPA primers according to the TwistDx guidelines [twistamp-assay-design-manual_rev1_v3.pdf (twistdx.co.uk)]. They are 30–35 bases long with 30–70% of GC content. The primers were designed to produce 100-bp to 400-bp *L. major*-specific amplicons. A fragment of at least 52 bp was maintained between the primer pairs to allow for internal probe design for lateral flow detection. The specificity of primer pairs was tested using RPA basic chemistry for the amplification and detection of products by the agarose gel electrophoresis.

In order to verify sequence conservation within *Leishmania* species and validate the designed RPA primers, for each selected target, we designed external PCR primers to survey conservation of the priming and probing sites through sequence analyzes of the targeted fragments across strains and species (Supplementary Table S2). Sequencing was performed on both strands using the BigDye Terminator v3.1 Cycle Sequencing Kit and an ABI 3500 sequencer (Applied Biosystems). The chromatograms were visualized and manually adjusted using the DNA Baser sequence assembler v4 program (Heracle BioSoft, www.DnaBaser.com). Multiple sequence alignment was performed using ClustalW, and the UPGMA tree was constructed using the Tree Builder tool, both integrated into Geneious 3.6.3 software.

### Setup of RPA basic assays

Reactions were set up following the TwistDx^™^ Basic RPA protocol; each reaction contained 29.5 μL rehydration buffer, 2.4 μL of each forward and reverse primers (10 μM), 11.2 μL dH_2_0, and 2 μL of *Leishmania* genomic DNA (20 ng/μl) for each reaction mix. A negative control (no template) reaction was included in each set of reactions. The reaction mix was then added to the dried RPA pellets containing a mix of enzymes and dNTPs, supplied as strips. Then, 2.5 μL of magnesium acetate (280 mM stock) was added to each lid making a total reaction volume of 50 μL. The strips were spun down and immediately placed into a thermoshaker (Neobiotech, France) for reaction initiation. Incubation was performed at 40°C for 30 min and under 600 rpm agitation, instead of manual shaking to ensure experiment reproducibility. Amplification products were next purified using the QIAquick PCR Purification Kit (Qiagen, Germany) and run on a 2% agarose gel. The primer pairs that showed positive *L. major-specific* amplification and negative results with *L. tropica* and *L. infantum*/*L. donovani* species were selected for further investigations.

### RPA using nfo chemistry coupled with LF detection assay

Multiple sequence alignment and experimental validation of the primers/probe sets were used to set up RPA under nfo chemistry to be coupled with LF detection assay. RPA reactions were performed in a 50 μL volume using an RAA amplification Kit—test strip method (Jiangsu Qitian Gene Biotechnology Co., Ltd. Wuxi, China). A master mix containing 420 nM of each forward and biotinylated reverse RPA primer, 120 nM FAM-tagged RPA probe, 25 μL of rehydration buffer, and DNase-free water was prepared and distributed into each reaction tube containing the dried enzyme pellet. Sample DNA (2 μL) was then added into each tube, and finally, a volume of magnesium acetate (280 mM) was pipetted into the tube lids and then centrifuged to allow initiation of the isothermal amplification reactions. Optimizations of RPA reaction were performed in order to determine the optimal conditions of reactions. Different magnesium acetate final concentrations ranging from 14 mM to 30 mM were tested. We also assessed different reaction times (10, 15, 20, 30, 35, and 40 min) and incubation temperatures (37, 39, 40, and 42°C).

Lateral flow detection was performed using generic 2 test lines PCRD cassettes (Abingdon Health, UK). Different dilution factors of the RPA products were tested involving 6, 10, 15, and 20 μL in a final volume of 90 μL with the supplied PCRD extraction buffer (provided with the kit by Abingdon Health). A volume of 75 μL was pipetted into the sample well of the PCRD test cassette and left for 5–10 min before it was imaged using a smartphone camera.

We performed the RPA and LF reactions in a unidirectional workflow using separated laboratory work areas for each step to prevent amplicon carryover contamination.

### Analytical sensitivity

To determine the analytical sensitivity, we used 10-fold serial dilutions of *L. major* DNA (R44) ranging from 20 ng/μl to 2 × 10^−5^ ng/μl where 2 μL was added in the reaction. Three independent experiments were performed to define the limit of detection of our assay.

### Performance evaluation of RPA-LF assays on clinical samples

A total of 86 DNAs extracted from skin lesion samples of 86 patients from the different study sites described above were tested blindly in order to evaluate our test performances. Sample status (positive/negative) was elaborated using the microscopy direct examination (DE) and ITS1 PCR (Schönian et al., 2003). In the case of samples with negative ITS1 PCR and positive RPA, DNA quality and/or the presence of PCR inhibitors was assessed by amplifying the human beta-globin gene (*β*-globin) as described by Bauer et al. (1991). In case of discordant results between the two reference tests (DE and ITS1 PCR), Lei70 PCR (Spanakos et al., 2002; Haouas et al., 2017) was used for confirmation of results. Species assignment was assessed using the ITS1 PCR-*HaeIII* RFLP (Schönian et al., 2003). Results were kept unknown from the user in order to eliminate the bias due to human behavior influenced by what is already known (Day and Altman, 2000).

## Results

### Selection of targets presenting potential priming and probing sites for *Leishmania major* DNA-specific amplification and detection

In total, we worked on 63 nuclear and 7 kinetoplast DNA targets presenting a percentage of pairwise similarity ranging between 94.7 and 76.3% among the different species of relevance: *L. major*, *L. infantum*, *L. donovani*, and *L. tropica*. These targets were also analyzed considering multiple sequence alignments to identify *L. major*-specific fragments as potential priming and probing sites, taking into account the recommendations for the design of primers and probes (size of segments and spacing), and the number of mismatches in these sites occurring between the different *Leishmania* species. In fact, available sequences of each target were retrieved from our data and the public databases aligned and the SNPs were identified for each species (*L. major, L. infantum/L. donovani*, and *L. tropica*) and manually screened for the criteria of specificity to *L. major* established above. We selected five DNA markers to target RPA development as the most interesting in terms of sequence specificity to *L. major*. Criteria for their selection were the presence of no less than four mismatches in at least one of the identified primers/probe sites when compared to the sequences of *L. tropica* and *L. infantum/L. donovani* strains and no more than one mismatch at the intraspecific level in each primer/probe site identified. We refined manually the design of the primers and probe sets. Positions of polymorphisms of the designed primers and probes are presented in Supplementary Table S3.

**Figure 1:**
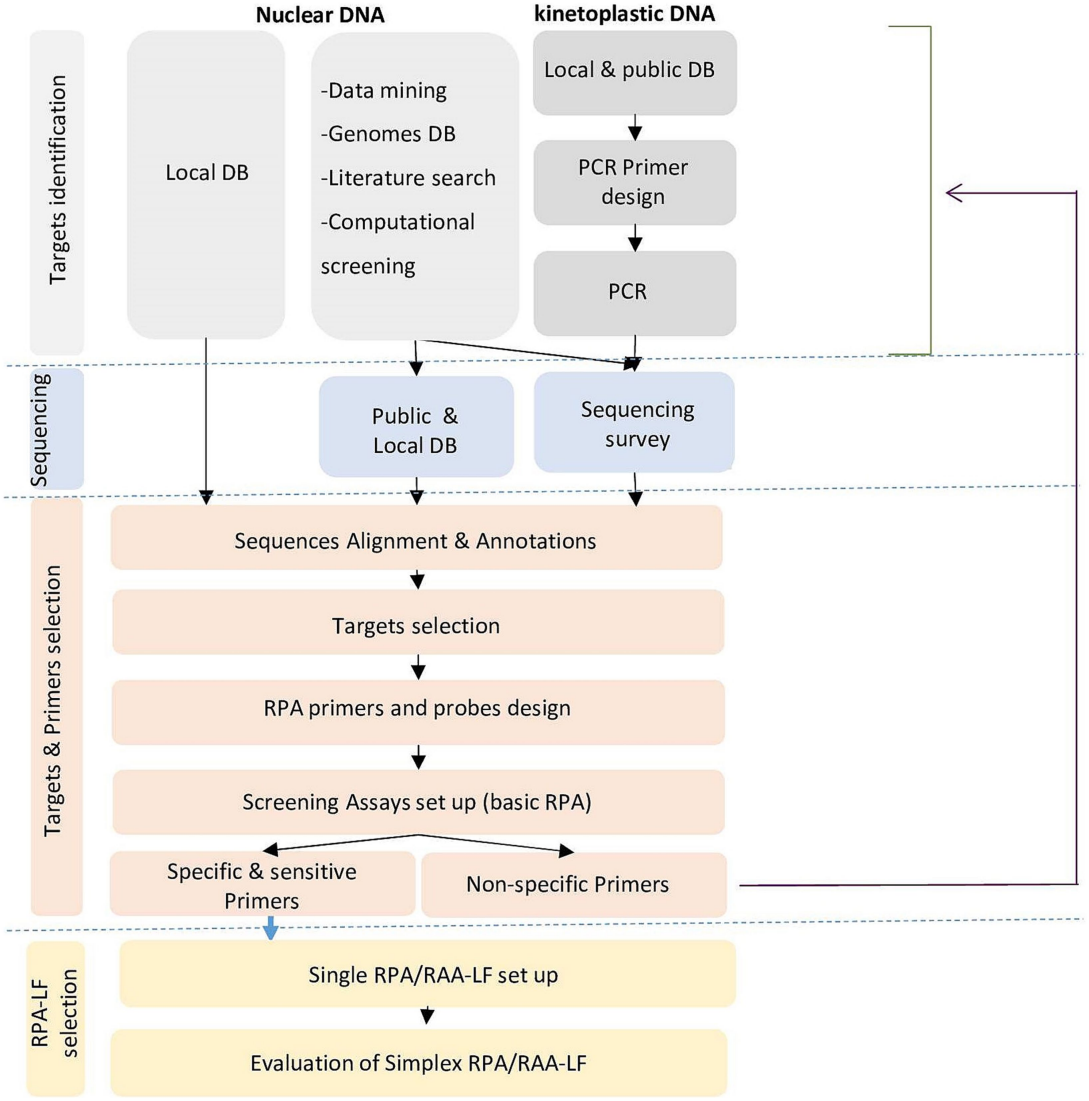
Decision tree for selection of targets and primers and their validation through an iterative approach for species-specific RPA/LF assays.

Given the number of targets investigated, to be cost-effective and productive, we have elaborated a decision-making tree for the selection of targets and primers and their validation through an iterative approach (Figure 1). Experimental validation of the primers was first done using the basic amplification kit for the detection of agarose gels. Then, the most interesting primers were labeled and tested for amplification including in the reaction mix the labeled internal probe (that acted as a semi-nested amplification primer) for more specific amplification, and sensitive and specific detection of the amplified products by lateral flow chromatography using a PCRD cassette.

### Validation of the RPA targets by sequencing analysis of the priming and probe sites across different *Leishmania* strains and species

To validate the primers and probe design, we surveyed the sequences selected for the occurrence of interspecies and intraspecies polymorphisms for five targeted markers (m30 (Genbank accession numbers PQ472465-PQ472485), m22 (Genbank accession numbers PQ538791-PQ538551 & PQ538541-538551), m27, m02 and m31) for which this information was missing for the studied *Leishmania* species (*L. major*, *L. infantum/L. donovani*, and *L. tropica*). The primers that showed intraspecies polymorphisms were rejected and other primers were designed using these sequence data, in regions conserved for *L. major*, but variable for the other *Leishmania* species. In total, we designed seven primer pairs and seven probes in five targets. Description of designed primers and probes is shown in Supplementary Table S4.

### Two-step establishment of recombinase-based amplification tests using the designed primers retains only one primer/probe set

**Figure 2:**
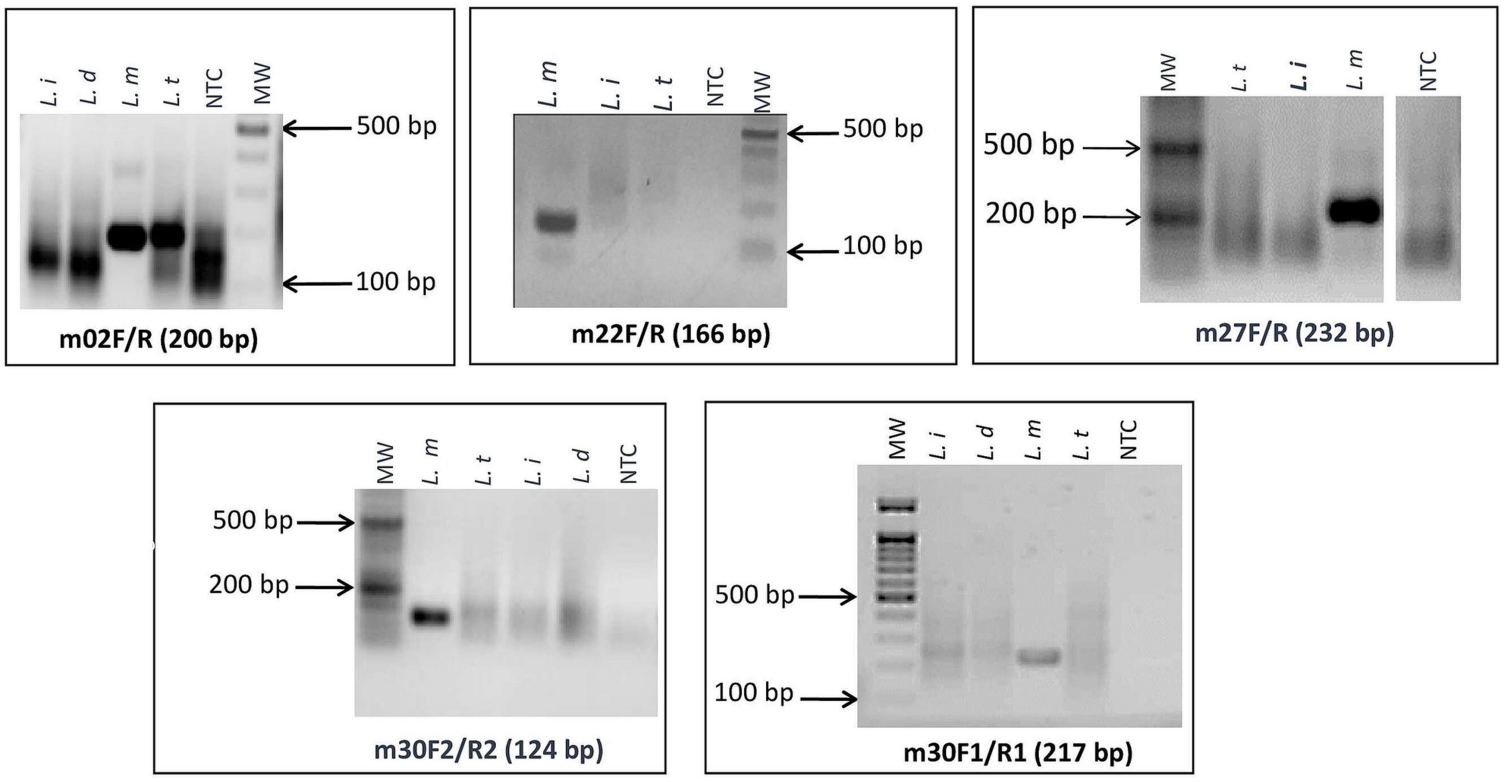
Screening assays and selection of *L. major* specific targets: agarose gel electrophoresis of targets that gave specific amplifications of DNA of *L. major* strains. *L. i: L. infantum; L. d: L. donovani; L. t: L. tropica; L. m: L. major*; NTC: no-template control; MW: 100-bp molecular weight.

As a first step, we screened the primers for specific amplification of *L. major* using RPA basic chemistry and agarose gel electrophoresis detection. Among the designed primers, we selected four primer pairs (m30_1, m30_2, m27, and m22) that gave specific amplifications of DNAs of *L. major* strains (Figure 2). The remaining primer sets showed cross-reactivity with other *Leishmania* species DNAs (m_02) or did not show amplification (m31 and m_30_3) with *L. major* DNA.

**Figure 3:**
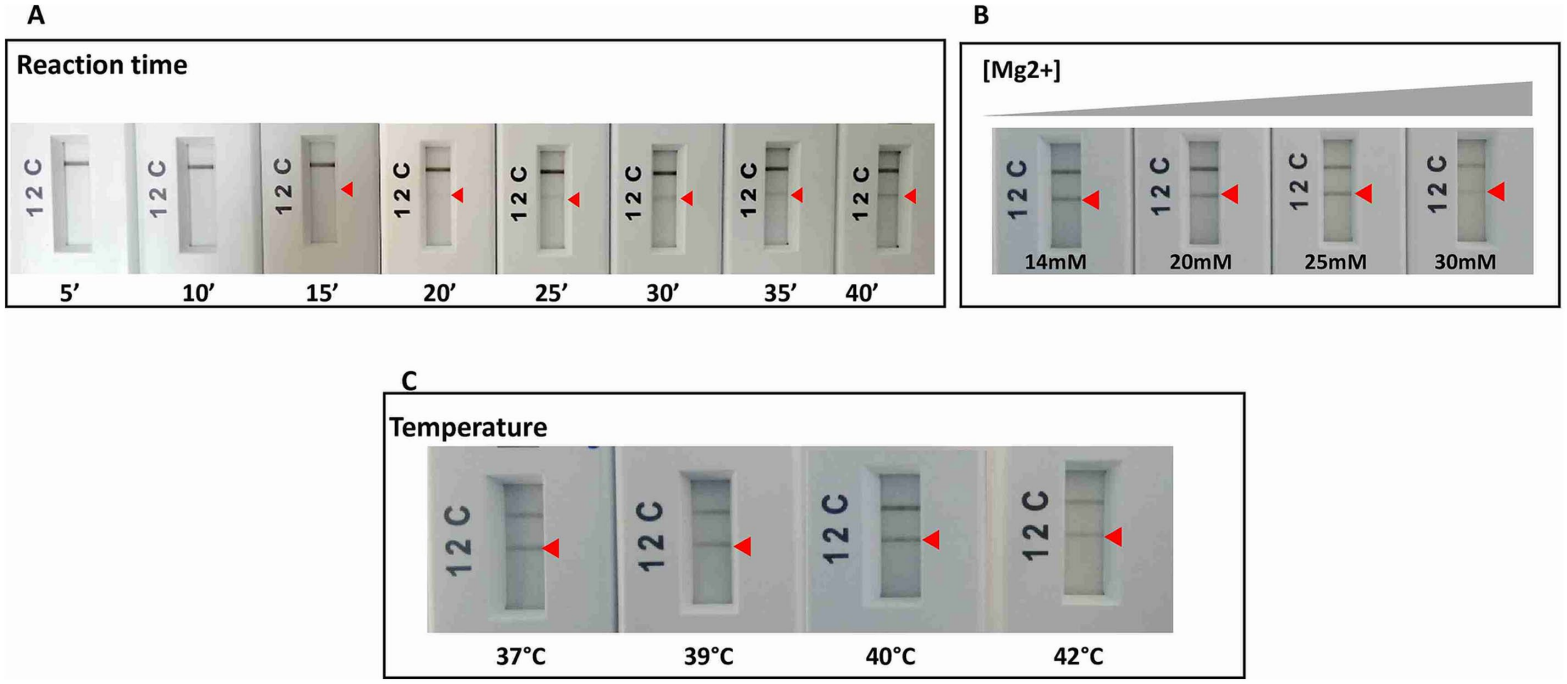
RPA reaction optimizations for the specific and sensitive detection of *L. major* species by varying: **(A)** time of reaction. **(B)** Magnesium concentration. **(C)** Temperature of reaction. Arrow: positive test line.

As a second step, the retained primers and their corresponding probes were tested using the RPA nfo chemistry Kit (TwistDx, UK), and when not anymore available on the market, the RAA test strip Kit (Qitian, China) was used to confirm the reproducibility of the results obtained. Amplified products were visualized on PCRD cassettes (Abingdon, UK). The selection criteria at this step were the specificity of results with the primers/probe sets used, and the absence of background noise with the no-template control in the PCRD test. Among the tested primers, only one (m22) gave the aimed result. Optimizations with this set allowed us to select incubation temperature of 40°C for 40 min and an Mg2+ concentration of 14 mM as optimal conditions for RPA or RAA (Figure 3). We selected 6 μL as amplification product input for the PCRD detection as higher reaction volumes have led to the appearance of background noise in the negative control tests.

**Figure 4:**
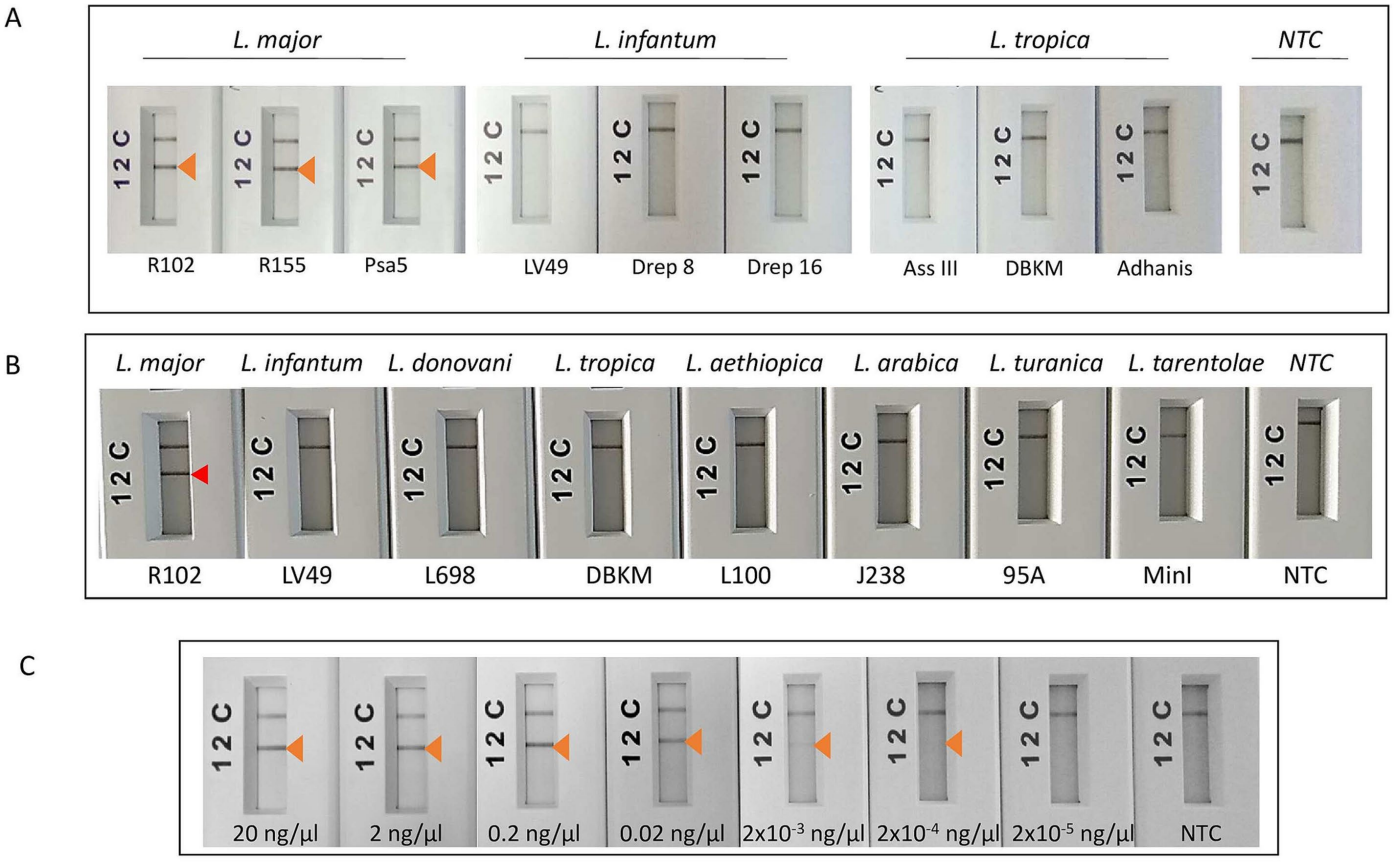
Test of the analytical specificity and sensitivity of m22-RPA/LF assays. **(A)** Test of species specificity of the selected m22-RPA/LF *L. major-*specific assay using different strains belonging to *L. major*, *L. infantum,* and *L. tropica* species. **(B)** Test of the reactivity of the m22-RPA/LF with other species. No cross-reactivity was observed with any of the species tested. The arrowhead indicates the positive test line. **(C)** Test of the analytical sensitivity of the selected m22-RPA/LF *L. major-*specific assay: We used serial dilutions of *L. major* strain DNA starting from 20 ng/μl to 2 × 10^−5^ ng/μl, using 2 μL as input for each reaction and 6 μL of the amplification product for LF detection. Arrow: Positive test line, NTC: no-template control. The test line corresponding to a DNA concentration of 2 × 10^−4^ ng/μL is visible to the naked eye but does not clearly appear in the smartphone photograph.

**Figure 5:**
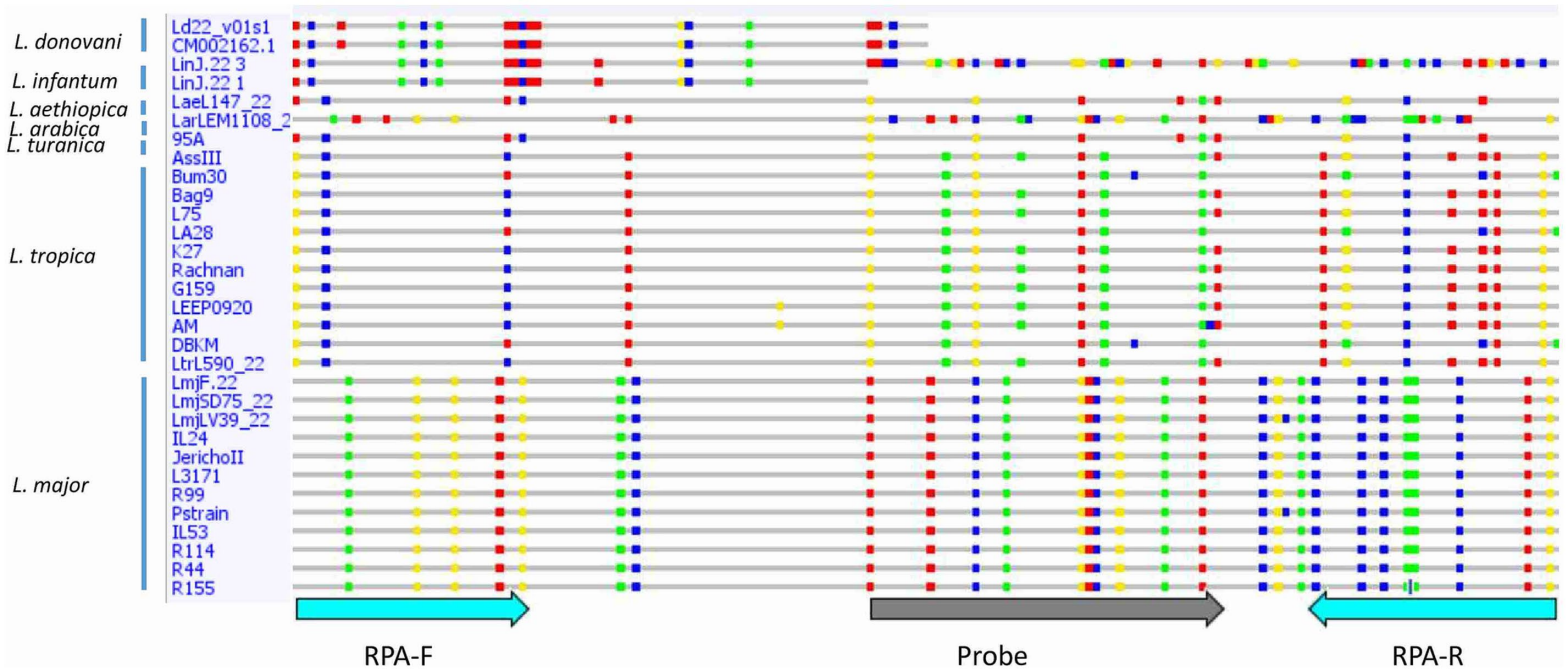
Sequence alignment of the selected target showing differences between *Leishmania* species/strains. Single nucleotide polymorphisms (SNPs) are shown in colored dots. RPA-F, Forward RPA primer; RPA-R, Reverse RPA primer.

**Figure 6:**
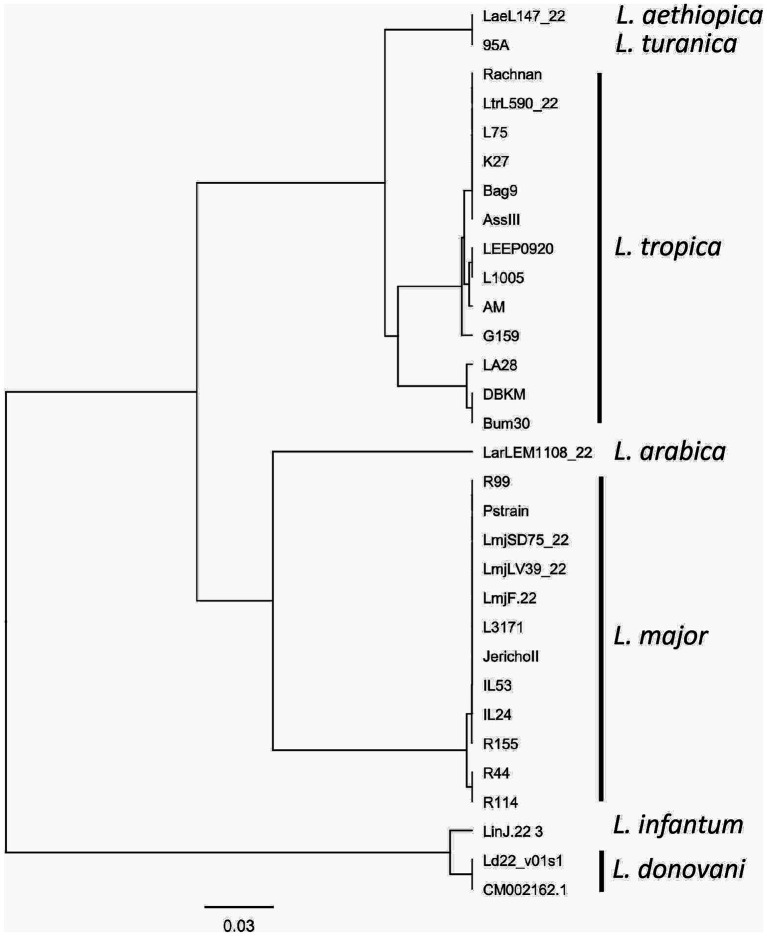
UPGMA phylogenetic tree based on the Jukes–Cantor genetic distance model. The tree illustrates the evolutionary relationships between the analyzed sequences, with branch lengths proportional to genetic distances. The Jukes–Cantor model was used to correct for multiple substitutions, ensuring an accurate representation of sequence divergence.

Consistent reactivity of the m22 primer set was assessed using a panel of DNAs of *Leishmania* species and strains. We have observed that our test yielded specific amplification of *Leishmania major* DNAs, consistently reacting with all the *L. major* representative strains DNA tested (*N* = 14) having different host and geographical origins. There was no cross-reactivity of the primers with *L. tropica* strains DNA (*N* = 8) nor with *L. infantum* strains DNA (*N* = 10) (Figure 4A) as demonstrated by the lack of amplified products for the DNAs of these species. Other species, including *L. donovani*, *L. aethiopica*, *L. turanica*, *L. arabica*, and the *Sauroleishmania L. tarentolae*, were also tested and showed no reaction with the m22 target (Figure 4B). This result is supported by the sequence alignment and analysis showing a pairwise similarity of less than 80% (Figure 5) and the UPGMA phylogenetic analysis (Figure 6).

The analytical limit of detection of the m22 set of primers was also tested using serial dilution of one *L. major* strain DNA starting from 20 ng/μl to 2 × 10^−5^ ng/μl, using 2 μL as input for each reaction and 6 μL of the amplification product for LF detection. We noticed that we were able to detect up to an input concentration of 2 × 10^−4^ ng/μl when we used RPA/LF analysis using the kits supplied from TwistDx (UK) or Qiagen (China) (Figure 4C), which here (4 × 10^−4^ ng) could correspond to 2.5 parasites considering that the average of diploid genome mass is 80 fg (Tupperwar et al., 2008).

### Application of the m22-RPA/LF assay to human cutaneous samples demonstrates its relevance for *Leishmania major* identification in CL lesions

To evaluate the use of the m22-RPA/LF assay for *L. major* detection in clinical samples, we tested 86 DNAs extracted from cutaneous scrapings and swabs taken from patients seeking diagnosis of cutaneous lesions. We used microscopic direct examination and ITS1 PCR for *Leishmania* detection and ITS1 PCR-*HaeIII* RFLP for identification of parasites as the classical methods. The samples provided blindly to the experimenter include infections due to *L. major* (*N* = 38), infections due to *L. infantum* (*N* = 6), infections due to *L. tropica* (*N* = 10), infections due to unknown species with positive direct examination and negative ITS1 PCR (*N* = 3) and negatives for *Leishmania* infections (*N* = 29) (Table 3).

**Table 3:** RPA-LF evaluation on cutaneous lesions’ samples.

Geographic origin	Patient	Patient Code	DE	ITS1 PCR	ITS1 PCR-*HaeIII* RFLP	m22-RPA-LF	Lei70 PCR	β-globin PCR
Sousse, Tunisia	1	PER340239	+	+	*L. major*	+		
	2	PER340247	−	+	*L. major*	+		
	3	PER340208	+	+	*L. major*	+		
	4	PER340213	+	+	*L. major*	+		
	5	PER340205	+	+	*L. major*	+		
	6	PER340203	+	+	*L. major*	+		
	7	PER340231	+	+	*L. major*	+		
	8	PER340204	+	**−**		+	+	+
	9	PER340241	−	−		−		
	10	PER340226	+	+	*L. major*	+		
	11	PER340217	−	−		−		
	12	PER340250	−	−		−		
	13	PER340219	+	+	*L. major*	+		
	14	PER340220	+	+	*L. major*	+		
	15	PER340242	+	+	*L. major*	+		
	16	PER340202	+	+	*L. major*	+		
	17	PER340214	+	+	*L. major*	+		
	18	PER340243	+	+	*L. major*	+		
	19	PER340225	−	−		−		
	20	PER340223	+	−		+	+	+
	21	PER340218	+	+	*L. major*	+		
	22	PER340273	+	+	*L. major*	+		
	23	PER340308	+	+	*L. major*	+		
	24	PER340269	−	−		−		
	25	PER340306	−	−		−		
	26	PER340301	−	−		−		
	27	PER340261	−	−		−		
	28	PER340349	−	−		−		
	29	PER340256	+	+	*L. tropica*	−		
	30	PER340336	−	−		−		
	31	PER340294	−	−		+	−	−
	32	PER340277	−	−		−		
	33	PER340257	−	−		−		
	34	PER340295	−	−		+	+	+
	35	PER340332	+	+	*L. major*	+		
	36	PER340322	+	+	*L. major*	+		
	37	PER340315	+	+	*L. major*	+		
	38	PER340290	+	+	*L. major*	+		
	39	PER340310	+	+	*L. major*	+		
	40	PER340282	−	−		−		
	41	PER340286	+	−		+	+	+
	42	PER340325	+	+	*L. major*	+		
	43	PER340284	+	+	*L. major*	+		
	44	PER340311	+	+	*L. major*	+		
	45	PER340267	+	+	*L. major*	+		
	46	PER340283	+	+	*L. infantum*	−		
	47	PER340345	+	+	*L. major*	+		
	48	PER340333	+	+	*L. major*	+		
	49	PER340337	−	−		+	+	+
Morocco	50	PER620249	+	+	*L. infantum*	−		
	51	PER620240	+	+	*L. infantum*	−		
	52	PER620229	+	+	*L. infantum*	−		
	53	PER620237	+	+	*L. infantum*	−		
	54	PER620216	+	+	*L. infantum*	−		
	55	PER620250	+	+	*L. tropica*	−		
	56	PER620234	+	+	*L. tropica*	−		
	57	PER620225	+	+	*L. tropica*	−		
	58	PER620220	+	+	*L. tropica*	−		
	59	PER620238	+	+	*L. tropica*	−		
	60	PER620156	+	+	*L. major*	+		
	61	PER620134	+	+	*L. major*	+		
	62	PER620155	+	+	*L. major*	+		
	63	PER620160	+	+	*L. major*	+		
	64	PER620122	+	+	*L. major*	+		
	65	PER620125	+	+	*L. major*	+		
	66	PER620163	+	+	*L. major*	+		
	67	PER620161	+	+	*L. major*	+		
	68	PER620143	+	+	*L. major*	+		
	69	PER620140	+	+	*L. major*	+		
	70	PER620223	+	+	*L. tropica*	−		
	71	PER620243	+	+	*L. tropica*	−		
	72	PER620211	+	+	*L. tropica*	−		
Tunis, Tunisia	73	PER570052	−	−		−		
	74	PER570100	−	−		−		
	75	PER570110	−	−		+	+	+
	76	PER570106	−	−		+	−	+ (faint)
	77	PER570104	−	−		+	−	+ (faint)
	78	PER570107	−	−		+	+	+
Lebanon	79	PER520118	+	+	*L. tropica*	−		
	80	PER520106	−	**−**	−	−		
	81	PER520110	−	−	−	−		
	82	PER520114	−	−	−	−		
	83	PER520109	+	−	−	−		
	84	PER520115	NA	−	−	−		
	85	PER520108	NA	−	−	−		
	86	PER520107	NA	**−**	−	−		

DE, Direct examination; NA, not available, +: positive, −: negative.

**Figure 7:**
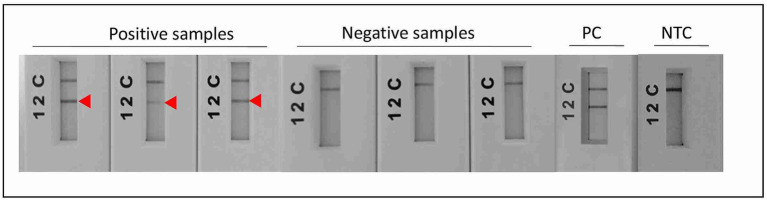
Test of *L. major-*specific target on CL samples using the m22-RPA/LF improved protocol. All *L. major-*infected patients were positive with our assay. PC: positive control, NTC: no-template control.

Using our assays, we were also able to detect 100% of *L. major* infections (38/38) within *L. major* CL patients having different parasite loads (Table 3; Figure 7), with 22 of them (58%) having a low parasite load (1–10 amastigotes/1,000 fields) (Table 3; Supplementary Table S1). The three patients with positive direct examination and negative ITS1 PCR results (PER340204, PER340223, and PER340286) were assigned to *L. major* when tested by our m22-RPA/LF tool. They were also positive when tested with the Lei70 PCR. No cross-species reactivity was seen when CL patients infected with *L. tropica* and *L. infantum* were tested. Among the 29 negative samples (DE-, ITS1-), seven gave positive results with our RPA/LF test. They were also tested with the generic Lei70 PCR (Spanakos et al., 2002; Haouas et al., 2017) to confirm their positivity. Among the seven samples, four were also positive with the Lei70 PCR (PER340295, PER340337, PER570110, and PER570107) (Supplementary Figure S1A). The remaining three samples were negative (PER340294, PER570106, and PER570104), with two showing a faint *β*-globin amplification band on upon agarose gel electrophoresis (Supplementary Figure S1B).

## Discussion

Cutaneous leishmaniases represent a wide range of cutaneous diseases characterized by their diverse clinical manifestations. These diseases are not fatal, but they are highly morbid and have a negative psychological impact on patients due to the lesions, the disfiguring scars, and subsequent stigma (Chahed et al., 2016; Pires et al., 2019).

CL are poverty-related infections that occur mainly in low-income and middle-income countries (Moya-Salazar et al., 2021) where health centers lack essential equipment for their diagnosis. Routinely *Leishmania* parasites from CL lesions are detected by direct microscopy examination, a laborious technique that lacks sensitivity. In addition, this technique is not able to identify *Leishmania* species, an important step that should be taken into consideration to guide CL treatment and patient management (Arevalo et al., 2007; Mosimann et al., 2013). This highlights the need for POC CL diagnostics to ensure healthcare equity for patients wherever they are.

The World Health Organization’s (WHO) roadmap to 2030, in terms of Research and Development on diagnostics needs to cover all NTDs, pointed to inadequate existing tools. Concurrently, in this study, we aimed to enhance access to CL diagnosis in endemic areas by developing an isothermal amplification coupled with lateral flow detection of *L. major*-infecting parasites. This test intends to improve CL diagnosis in low-resource settings where infections due to *L. major* are predominant like, for instance, in North African countries, or highly endemic like in other countries in Africa, the Middle East, and Asia. According to WHO, an ideal POC test should be affordable, sensitive, specific, user-friendly, and equipment-free and delivered to patients (ASSURED) (Kosack et al., 2017). In this study, we provided a proof of concept in a laboratory environment that RPA/LF assays are suitable for the detection and identification of *L. major* in clinical samples. The method has several inherent advantages; it is simple, rapidly processed, and needs low-cost basic laboratory equipment. The time to result delivery is 1 h, and it includes amplification, detection, and identification of *L. major* parasites. The RPA ready-to-use lyophilized reagents provided in a single tube make the reactions simple and rapid to perform without any expertise needed. In addition, the design of the assay is simpler than LAMP, an isothermal amplification technology that requires four to six primer pairs. It is also known that RPA tolerates PCR inhibitors (Kersting et al., 2014; Krõlov et al., 2014; Rosser et al., 2015; Tan et al., 2022), so it could be coupled to minimally processed and simplified methods for DNA extraction (Saldarriaga et al., 2016; Moore and Jaykus, 2017; Munawar et al., 2020). All these advantages are favoring factors for the widespread adoption of the RPA/LF platform to support POC diagnosis of cutaneous leishmaniases.

RPA technology was used for the detection of diverse pathogens including bacteria, viruses, and parasites (Kersting et al., 2014; Krõlov et al., 2014; Rosser et al., 2015; Munawar et al., 2020). During the last decade, many studies reporting RPA assays for the detection of *Leishmania* parasites were published (Mondal et al., 2016; Saldarriaga et al., 2016; Gunaratna et al., 2018; Cossio et al., 2021; Ghosh et al., 2021; Khan et al., 2021; Mesa et al., 2021). These assays use protocols with different end-point readouts including LF (Saldarriaga et al., 2016) or real-time portable tubes scanner (Mondal et al., 2016) to detect, namely, *L. donovani* (Mondal et al., 2016) and *L. viannia* parasites (Cossio et al., 2021; Travi et al., 2021; Saldarriaga et al., 2016). A recent study evaluated a mobile laboratory suitcase that includes an RPA method for the detection of *L. donovani* parasites in VL and PKDL cases in a multi-country phase 2 study and demonstrated the accuracy of this test for VL and PKDL diagnosis (Ghosh et al., 2021). However, investigations using RPA technology for CL diagnosis were lacking. Thus, we intended to develop a CL RPA test that could be used as a screening assay to complement other DNA methods in countries/regions where *L. major* is highly prevalent. To our knowledge, this is the first report describing an RPA test that specifically detects *L. major* parasites. It is known that *L. major* infections occur mostly in rural arid areas of North Africa and the Middle East where it frequently causes epidemics (Gradoni, 2017). *L. major* is also a widespread species across Africa and Asia. All these transmission areas typically lack healthcare infrastructure; point-of-care diagnostics that could be deployed as portable solutions, relying on basic equipment, is therefore needed for timely patient management and early alert on emergence. However, it is clear that a cost-effectiveness analysis of the RPA/LF assays is requested as it is another crucial factor for diagnostics scalability and implementation in resource-limited settings (Aerts et al., 2019).

In this study, we report on the development of an *L. major* RPA/LF assay in which we encountered two major challenges. The first one was the design of primers/probes that selectively detect *L. major* strains. RPA is known to tolerate mismatches in priming sites (Boyle et al., 2013; Daher et al., 2015). However, Boyle et al. (2013) were unable to amplify one HIV variant using an RPA reaction because of the presence of six nucleotide changes and one insertion in the primer/probe binding sites (Boyle et al., 2013). Thus, our strategy has relied on selecting an extensive number of mismatches between *L. major* and other *Leishmania* DNAs, flanking our target fragment, to maximize the chances to amplify selectively *L. major* DNA. In fact, the finally retained assay, m22-RPA, targeted a non-coding fragment in *L. major*, originally identified as an *L. infantum-*specific coding gene. In *L. major*, the selected primers/probe sites include five mismatches in the forward primer, nine in the reverse one, and nine in the probe when compared to *L. infantum* or *L. tropica* DNA sequences. These mismatches are specific to *L. major* strains and are present in all sequenced *L. major* DNA fragments amplified covering strains from North Africa, Asia, and the Middle East and in the sequences extracted from the database of two Middle Eastern (Friedlin and LV39) and one African strains (SD75). This confirms the relevance of the primers and probe set for a consistent reactivity within the species. In addition, in the cases of *L. aethiopica* and *L. donovani*, the priming site for the reverse primer was absent, while for the other species, the priming sites exhibited significant divergence from the *L. major* sequence, with less than 80% similarity. The UPGMA tree is consistent with these results, further highlighting the taxonomic potential of the selected m22 target. It was further confirmed experimentally with the panel of *Leishmania* species and strains tested. In addition, our results corroborated that efficient RPA reaction leading to species-specific amplification may depend on the number and positions of mismatches (Daher et al., 2015). The type of nucleotide involved in the mismatch was also shown to significantly impact RPA reaction efficiency, with primers’ terminal cytosine–thymine and guanine–adenine mismatches having the most detrimental effects (Higgins et al., 2022).

The second challenge was the formation of primers and probe/primer dimers during the RPA reaction. Indeed, the length of the primers/probes together with the low reaction temperature contribute to primer dimers noise that could appear in the LF-negative tests and lead to confusing results. Some studies opted for a post-RPA heating step (Liu et al., 2017) or used self-avoiding molecular recognition systems (SAMRS) oligonucleotides (Sharma et al., 2014) to overcome this noise issue. In our case, we have aimed at delivering simple assays performing at a single and low temperature. Therefore, to resolve this problem, we have designed and tested many primers to produce reliable assays and clear negative results in the LF device with the negative samples and the control samples without template DNA. We also carefully selected the oligonucleotides after checking their *in silico* properties (secondary structure, GC content, and repeats) but also their specificity, by performing a blast analysis with all DNA sequences available in NCBI and TritrypDB databases. It was reported that the non-specific background DNA could interfere with RPA reactions and diminish its efficiency depending on the primers/probes used and their level of interaction with the background DNA (Rohrman and Richards-Kortum, 2015). In this study, we used CL samples during the whole process of development in order to verify potential cross-reactivity of the tests under development with the human DNA and other pathogens that could be present in cutaneous lesions. Then, we extended the study by evaluating our assays on a larger number of DNAs extracted from cutaneous samples from different geographic origins in countries from MENA, including Tunisia, Morocco, and Lebanon. We used harmonized protocols for sampling. Tunisian, and Moroccan teams succeeded in using lesions scrapping for the detection and identification of parasites. However, the Lebanese team performed DNA extraction on swabs taken on the scrapped lesions. As standard techniques for RPA assay validation, we used the combination of direct examination and ITS1 PCR for the detection of *Leishmania* parasites, and ITS1 PCR-*HaeIII* RFLP for parasites identification. Our *L. major* RPA-/LF-specific test was able to detect 100% of *L. major* infections within CL patients, independently of the parasite load estimated by microscopy on the slides. Importantly, the test performed well in all cases where the parasite load was low, highlighting the relevance of the assay to support diagnosis as a complement or alternative to microscopy. Among the negative samples, seven showed positive results. These samples belong to patients with CL-like clinical features.

Four of them (PER340295, PER340337, PER570110, and PER570107) showed positive results with the generic Lei70 PCR. These samples are considered true positive. The three remaining samples (PER340294, PER570106, and PER570104) were negative with the generic Lei70 PCR. Two of these patients (PER570106 and PER570104) exhibited a faint *β*-globin amplification band, suggesting poor DNA quality or the presence of PCR inhibitors in the extracted DNA. Unlike PCR, however, it is well documented that a wide range of inhibitors in the reaction can be tolerated, making it a more robust method for challenging samples (Kersting et al., 2014; Krõlov et al., 2014; Rosser et al., 2015; Tan et al., 2022). For patient PER340294, the parasite load in the lesion sampled may be very low and therefore not detectable by the standard molecular tests used nor by microscopic direct examination. Two hypotheses could be stated in this case. The first one is that these samples are false positives, and then, our test is not 100% specific (Sp = 88%). The second hypothesis is that these samples are true positive, and then, our test is more sensitive than the used reference diagnosis tests.

Given the ongoing climatic change impacting the complex epidemiology of Leishmaniases, particularly in Africa and the Middle East, there is a growing risk of the emergence of new *Leishmania* species in areas where *L. major* is prevalent, which may lead to co-sympatry, mixed infections, and generation of interspecific hybrids. Expansion of *L. major* into other areas so far free of leishmaniases could also occur. Accurate diagnosis of leishmaniases, *L. major* cases in particular, is therefore increasingly important. In instances where the developed test is negative for *L. major*, additional tests should be considered to confirm whether the sample is truly negative or is infected with another *Leishmania* species. It was shown that using multiple diagnostics tests achieves a greater specificity than any single test (Gass, 2020). In addition, establishing an algorithm to guide diagnosis should be envisaged as it may depend on the tested population, disease prevalence, and specimens (Hurt et al., 2017). Developing a simple and rapid molecular test to screen *L. major* CL infections in regions where this species is predominant is in line with such a purpose.

Expanding the RPA-LF platform to encompass CL cases due to other *Leishmania* species of relevance is in our perspectives, permitting us to detect and identify all CL cases including those that are caused by mixed infections or by natural interspecific hybrids such as the ones recently reported in Ethiopia (Hadermann et al., 2023). As a *Leishmania infantum* RPA/LF assay has already been developed and validated for the diagnosis of visceral leishmaniasis (Castellanos-Gonzalez et al., 2015), it would be worthwhile to explore its potential application for cutaneous leishmaniasis caused by this species, and the closely related *L. donovani*. Furthermore, as a future direction, the development of *L. tropica* and *L. aethiopica* species-specific RPA/LF assays could address a critical diagnostics gap, particularly in the African and Middle Eastern region, where *L. major*, *L. tropica*, *L. aethiopica,* and *L. infantum/L. donovani* are the most prevalent species causing cutaneous leishmaniases. Expanding the RPA/LF platform to cover these key species would greatly enhance diagnostics capacity in these endemic areas. Moreover, incorporating into the assays an internal control for human DNA compatible with an LF detection will enhance the reliability of the test by verifying its validity and confirming negative results.

Additional developments to our current RPA/LF assay and future platform should be considered, bringing them closer to the point of care by shortening further the timeline of the workflow, reducing the costs, and automating the readouts. Indeed, the current DNA extraction step presents a challenge for implementing the test in resource-limited settings and its scalability. Therefore, the integration of an upstream simple DNA extraction and a less costly method within the workflow, as described by Mondal et al. (2016), will enhance the feasibility and usability of the test at the point of care. Moreover, developing and using a lateral flow smartphone reader application could significantly enhance test interpretation and minimize observer bias. This technology is particularly valuable for resolving ambiguities associated with faint bands and overcoming challenges like the one we encountered during our evaluation of the analytical limit of detection of assay.

## Conclusion

We have developed a platform of an *L. major*-specific and sensitive RPA/LF assays. Through this proof-of-concept study, we have provided evidence of the efficacy of our RPA-LF assays for the detection and identification of *L. major* species in CL lesions. Our tests could be expanded/used to complement other recombinase-based amplifications to detect and identify *Leishmania* parasites in a point-of-need context.

## Data Availability

The datasets presented in this study can be found in the manuscript. The DNA sequences can be found in online databases with the names of the database and accession number(s) found in the article.
