# Supplementary figures and images for: Recombinase-based amplification coupled with lateral flow chromatography for the specific and sensitive detection and identification of Leishmania major in cutaneous leishmaniasis patients
Source: Front Microbiol. 2025 Jan 27;15:1514684. doi: 10.3389/fmicb.2024.1514684 (PMC11807989; doi:10.3389/fmicb.2024.1514684)

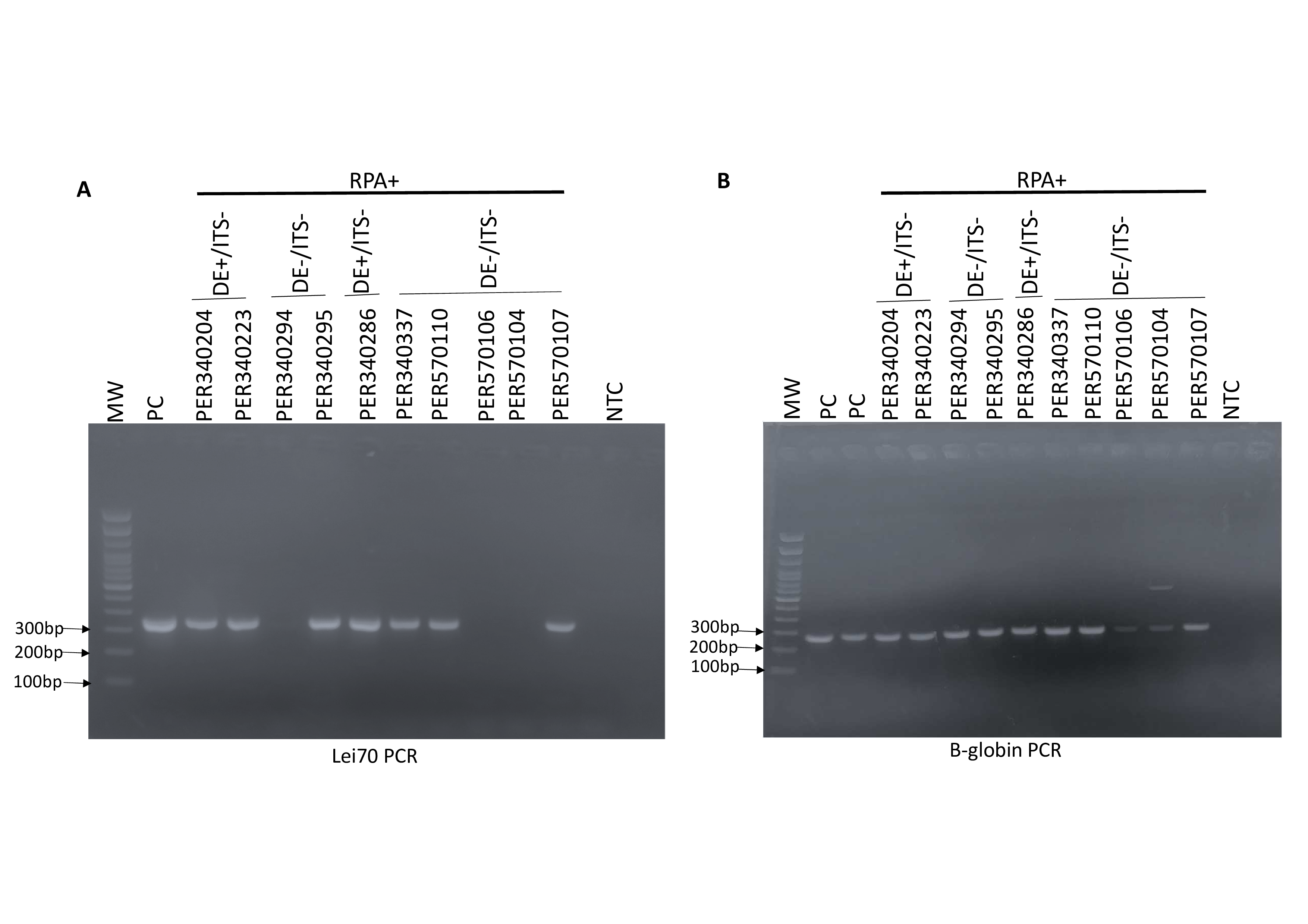

Supplement: Supplementary file 1 [file Image_1.TIFF]
